# Positive effects of online games on the growth of college students: A qualitative study from China

**DOI:** 10.3389/fpsyg.2023.1008211

**Published:** 2023-02-24

**Authors:** Feiyue Li, Di Zhang, Suowei Wu, Rui Zhou, Chaoqun Dong, Jingjing Zhang

**Affiliations:** ^1^School of Nursing, Wenzhou Medical University, Wenzhou, China; ^2^School of Mental Health, Wenzhou Medical University, Wenzhou, China

**Keywords:** college students, online games, Glory of Kings, personal growth, social life, academic performance, qualitative study

## Abstract

**Objectives:**

This study aimed to explore the positive effects of online games on college students’ psychological demands and individual growth.

**Methods:**

A qualitative study design was carried out in September 2021. Semi-structured, in-depth, and individual interviews were conducted with a purposive sample of 20 undergraduates who played the online game “Glory of Kings” from six universities. Thematic analysis was employed to explore the positive features caused by “Glory of Kings”.

**Results:**

College students reported three positive effects of online games, namely, satisfying the need for personal growth, meeting the requirement of social life and promoting academic performance.

**Conclusion:**

College educators and families should take advantage of the positive effect of online games to guide college students to use online games reasonably.

## 1. Introduction

According to the survey data of the China Internet Network Information Center (CNNIC), by the end of December 2021, the number of netizens in China reached 1.032 billion, of which the number of online game users reached 554 million (CNNIC, 2022). The number of game apps reached 709,000, accounting for 28.2% of all apps (CNNIC, 2022). Online games include massive, multiplayer, online role-playing games (MMORPGs), first-person shooter (FPS), real-time strategy (RTS) games, and other games (Park et al., 2016). MOBA (Multiplayer Online Battle Arena) game, a subgenre of RTS games where two teams of five players usually play against each other (Mora-Cantallops and Sicilia, 2018b), is one of the most popular online games in China because of its competitive, interactive, and simple operating characteristics. Specifically, “Glory of Kings,” as a MOBA game, is listed at the top of the Chinese mobile game charts for contemporary college students (Huang, 2021).

Online games are criticized by educators because many students invest a lot of time, money, and energy into games, which seriously affects their academic studies, social interaction, and physical and mental health, and ultimately leads to the tragedy of online game addiction (Freeman, 2008; Kuss and Griffiths, 2012; Blinka and Mikuška, 2014; Xu et al., 2017). Online game addiction, as one of the most serious behavioral addictions (Lopez-Fernandez, 2018), could cause a series of physical and mental problems, such as poor sleep, depression, anxiety, or even death (Ferguson et al., 2011; Kuss and Griffiths, 2012; Wei et al., 2012). Meanwhile, parents’ opposition to online games can also be observed in family education. Studies have shown that the more addicted adolescents are to online games, the worse their parents’ attitude toward online games (Jeong and Kim, 2011). Many adolescents who love playing online games face restrictions or prohibitions from their parents over the time spent on the Internet or other ways. For example, in a study of 2,021 adolescents, parental restrictions were 1.9 times higher among adolescents who were overly addicted to online games than among other adolescents (Wu et al., 2016). Parents who do not show enough attention to their children promptly can lead to children using online games to divert negative family-related emotions (Xu et al., 2021).

However, it is worth noting that the majority of studies focus on the negative effects of online games (Lo et al., 2005; Ng and Wiemer-Hastings, 2005; Yc, 2006; Smyth, 2007; Li and Wang, 2013), while positive effects are neglected. In fact, playing online games at a moderate level could be beneficial to players’ personal psychological growth and interpersonal relationships (Ko et al., 2005; Yee, 2006b; Granic et al., 2014). In terms of emotional experience, existing research on the emotional impact of online games suggests that they have the potential to reduce depression, stress and obtain happiness (Wu and Liu, 2007; Ari et al., 2020; Pine et al., 2020). In the process of psychological development, college students’ cognitive, memory, and other mental skills are proved to be enhanced by online games (Boot et al., 2008; Glass et al., 2013; Oei and Patterson, 2013). There is evidence that games have the potential to provoke thought about the player’s personal development and ideals and increase the sense of self-realization if the players have strong ability in online games (Nuangjumnong, 2014; Bopp et al., 2016; Mora-Cantallops and Sicilia, 2018a). In social life, online games also establish the value of social connectedness and enhance the sense of interaction (McClelland et al., 2011; Snodgrass et al., 2011; Oliver et al., 2016). Sublette and Mullan (2010, p. 20) argue that through online games “socialization may just shift in focus: while real-world relationships eroded for some players.” It is further proposed that intimacy in games will also extend to offline real life, and shared game experience will reinforce offline communication (Kim and Kim, 2017; Lai and Fung, 2019). MOBA games focus on personality development and teamwork in battle (Yang et al., 2014; Mora-Cantallops and Sicilia, 2018b). In other words, electronic space expands social communication to the virtual field (Yee, 2006a) and increases the team cooperation consciousness, leading to diverse communication ways. Besides, in terms of learning, online games are proven to help students engage in learning activities (Iaremenko, 2017; Schenk et al., 2017; Calvo-Ferrer and Belda-Medina, 2021).

Existing research confirms that the academic performance and satisfaction of Chinese college students positively impact on the continued use of the “Glory of Kings” and promote the reconstruction of the player’s social image (Chen and Chang, 2020). As mentioned above, previous studies have examined various positive aspects of online games, but the studies are based on foreign cultural contexts. Research subjects from different cultural backgrounds may have different perceptions and influences on online games, so it is culturally significant to study the positive influence of online games on Chinese college students. In other words, whether they can apply to the Chinese culture and environment or whether similar conclusions can be drawn among the Chinese college gamer population, has not been verified. Moreover, although “Glory of Kings” is widely concerned and popular among college students, few scholars in China try to evaluate the positive impact of “Glory of Kings” on college students. The design and development of “Glory of Kings” have distinctive Chinese cultural characteristics, therefore, the choice of “Glory of Kings” as a carrier for the study has cultural significance. Secondly, the widespread usage of “Glory of Kings” among college students provides universality for the study. Third, since most of the existing studies are quantitative, qualitative research can enrich the existing research results, explore new experiences, and make relevant suggestions for higher education and family education. In conclusion, given the current popularity and influence of “Glory of Kings” in China, an in-depth study of Chinese college student players was conducted using it as a medium for qualitative research.

## 2. Materials and methods

### 2.1. Participants

A purposive sampling method was adopted to select college students who were “Glory of Kings” players from six universities or colleges in Zhejiang Province. Inclusion criteria: (1) college students; (2) playing the “Glory of Kings” game for more than 1 year; (3) have participated in the “Glory of Kings” game in the ranking tournament; and (4) informed consent and voluntary participation. Exclusion criteria: (1) college students suspended from school due to physical problems; (2) students who have a medical history of mental illness or psychiatric disorders and who were screened as having mental problems in the students’ general psychological test. The sample size was determined based on the principle of theoretical saturation. Interviews were conducted until reached theoretical saturation—that is, when the 18th participants did not provide new insights, and two more interviews were conducted to verify if new information would emerge. In the end, no new ideas were found to emerge making the sample size appropriate for this study. Among 23 students who were invited to participate, one student refused to participate due to lack of interest, and two persons were unable to participate due to time conflicts. Eventually, 20 college students (13 males and 7 females) were interviewed. Their ages ranged from 18 to 21 years, and their experience in playing the “Glory of Kings” game ranged from 2 to 6 years. The detailed information of participants is shown in Table 1.

**TABLE 1:** The basic information of the “Glory of Kings” player interviewees.

No	Age	Year	Major	Years of playing “Glory of Kings” (year)	Academic performance ranking in semester 1	Academic performance ranking in semester 2
F1	19	Year 2	Pharmacy	4	13/30	10/30
F2	19	Year 2	Traditional Chinese Medicine	3	10/50	13/50
M1	20	Year 3	Management of public services	2	45/78	39/78
M2	20	Year 3	Marketing	4	5/50	4/50
F3	18	Year 1	Statistics	5	23/123	27/123
F4	19	Year 1	Chemistry	5	45/69	43/69
F5	19	Year 2	Medicine	2	17/45	17/45
M3	19	Year 2	Philosophy	6	45/58	50/58
M4	21	Year 4	Law	6	30/59	31/59
M5	20	Year 3	Accounting	6	34/50	29/50
M6	19	Year 2	Advertising	6	46/68	42/68
M7	19	Year 2	Traditional Chinese Medicine	6	14/30	12/30
M8	18	Year 1	Pharmacy	5	7/34	7/34
F6	20	Year 3	Traditional Chinese Medicine	5	38/79	35/79
F7	19	Year 2	Journalism	4	17/30	18/30
M9	20	Year 3	Business Management	6	16/36	21/36
M10	20	Year 3	Physics	4	45/60	35/60
M11	18	Year 1	Biotechnology	5	56/68	50/68
M12	18	Year 1	Clinical Pharmacy	6	39/68	30/68
M13	19	Year 2	Psychology	2	45/56	42/56

M, male; F, female. Years of playing “Glory of Kings” is calculated from the first time start playing the game.

### 2.2. Data collection

Participants were recruited in September 2021 with the assistance of the university’s gaming societies. The gaming societies presented the study recruitment information to members *via* social media. Participation was voluntary, and no incentive was offered for participation. The demographic information was collected before the interview, and the GPA information was collected at the beginning and end of the semester. The pilot-tested, semi-structured interviews were conducted using pre-determined, open-ended questions. The interviewers were conducted by two senior undergraduate students who had acquired knowledge of psychology, interpersonal communication, and qualitative research through relevant training prior to conducting this study. When conducting the interviews, they would follow a unified syllabus and agree on the follow-up questions. The interviews were conducted in a combination of online and offline formats. For participants located in Wenzhou City, interviews were conducted face-to-face in a meeting room at Wenzhou Medical University, where the environment was quiet and undisturbed. For participants located outside of Wenzhou City, online video interviews were conducted using social media. In addition to questions on demographic information, the interview syllabus was as follows:

1. Why do you play games?
2. How do you feel about being a player in the game of “Glory of Kings?”
3. What do you expect to gain from playing the game of “Glory of Kings?” And what do you actually gain from the game?
4. What is your experience with playing the game? And what experiences do you find enjoyable?
5. What are the best and worst things you think happen in the game?
6. What personal changes do you think have occurred after playing the game?
7. Is there anything else you would like to talk about on this topic?

All interviews were conducted at the time most convenient to the participants, and the interview schedule was determined 1 day in advance. Before the interview, participants were told about the procedures, such as how long the interview would last, the topics to be discussed, and permission to record the interview. Each interviewee lasted for approximately 30–40 min. All interviews were audiotaped and transcribed within 24 h after the interview. Two researchers independently completed and checked transcription to reduce personal biases.

### 2.3. Data analysis

Inductive thematic analysis was used to analyze the data (Braun and Clarke, 2006) and data were analyzed using qualitative analysis software NVivo 10. The steps were as follows: (1) transcript reading, preliminary coding, and note-taking; (2) developing final codes by reading and rereading the transcripts to identify patterns and themes; (3) developing a thematic mind map; (4) defining and naming themes and sub-themes, and (5) preparing the final report with an analysis of the selected fragments. Data were analyzed by two researchers to ensure reliability, when other researchers examined and validated the data, codes, and analyses by holding regular research team meetings. Textual information was discussed by the researchers several times until a consensus was reached. In addition, the results of the preliminary analysis were shared with the participants for their reviews and comments. The analysis was done in Chinese and the quotes were then translated into English and checked by a native translator who was not involved with the data collection (see Table 2 for an example of the process).

**TABLE 2:** Thematic analysis of transcribed data from interviews with 20 people with the online game “Glory of Kings”.

Meaning unit	Condensed meaning unit	Subtheme	Theme
I went through a particularly bad period last semester. The heavy pressure of study and life made me breathless. Later, I fell in love with “Glory of Kings,” and sometimes I even stayed up late to play games, which I know it is a bad habit. However, when I was addicted to games temporarily, my pressure was released, which provides enough energy and enthusiasm to face difficulties.	Noticing negative emotion and trying to release it	Relieve stress and achieve happiness	Meeting the need for personal growth
I like to play alone in tournaments and always rush to the front alone, which leads me to always lose the game. Gradually, I started to think about the reason, until I realized that I lacked a sense of the big picture and cooperation. Since then, I’ve made a conscious effort to work with my team on offense, which provides me with a better chance of victory. In life, I also recognized similar shortcomings and corrected them.	Be aware of some details that are overlooked in real life	Overcome the shortcomings and gain self-awareness	
In real life, people around me pay special attention to GPA. Sometimes I feel inferior because of my poor academic performance, and my classmates often treat me with colored glasses… I’m not sure if this is because I’m overreacting, I feel that way from time to time. However, online games provide a whole new world. Ranking as a king in the game, many players are willing to team up with me, which gives me a sense of accomplishment and brings back the confidence I lost in real life.	He gets positive feelings that he can’t get in the real world	Gain achievement and self-realization	

Examples of meaning units, condensed meaning units, and subthemes and theme.

### 2.4. Ethical considerations

The studies involving human participants were reviewed and approved by the Ethics Committee of Wenzhou Medical University (2022-028). This study complies with the Helsinki Declaration’s guidelines. After obtaining both verbal and written information about the study, the participants signed informed consent. Written informed consent was obtained from the individuals for the participation in this study and the publication of any potentially identifiable images or data included in this article, and the data was password-protected and encrypted. Each participant was assigned a coding reference, which referred to the participant’s number and gender (i.e., M3 refers to respondent 3, Male gender). During the interviews, the researchers treated the participants with respect, listened intently, and responded to their questions.

## 3. Results

Three themes were identified from the data material, namely, meeting the college student’s personal growth, satisfying the social needs of college students, and promoting academic performance.

### 3.1. Meeting the need for personal growth

#### 3.1.1. Relieve stress and achieve happiness

Online games, because of the characteristics of confrontation, entertainment and challenge, make it a way for college students to vent their emotions. Most interviewees believe that the fast pace of university brings them great study pressure. Also, college students of different grades report different sources of stress, such as pressure from parents, peers, academics, etc. However, no matter what kind of stress leads to negative emotions, they can be relieved through entertainment games. Following are typical quotes from gamers who said that the game can relieve negative emotions and bring positive feelings.

Game is not a necessity, but I would occasionally indulge in it. I like to live happily in virtual games (M5, 20-year-old).

When encounter troubles, I choose to play a few rounds of games. Although the pleasure brought by the game is temporary, it could fade away the troubles (M1, 20-year-old).

I went through a particularly bad period last semester… Later, I fell in love with “Glory of Kings,” and sometimes I even stayed up late to play games… However, when I was addicted to games temporarily, my pressure was released, which provides enough energy and enthusiasm to face difficulties (F7, 19-year-old).

#### 3.1.2. Overcome the shortcomings and gain self-awareness

Due to the nature of “Glory of Kings,” it provides a platform for college students to fully enhance their consciousness. The characteristics of the five different roles in this game can also further prompt players to recognize their shortcomings and provide a reference for players to understand themselves. Players whose self-awareness is not clear enough get a clearer understanding through the game, and players whose self-perception is vague or even sometimes wrong get a chance to correct it. Following are typical quotes from gamers who have better self-awareness.

Gradually, I started to think about the reason, until I realized that I lacked a sense of the big picture and cooperation. Since then, I’ve made a conscious effort to work with my team on offense, which provides me with a better chance of victory. In life, I also recognized similar shortcomings and corrected them (F1, 19-year-old).

I prefer to play the role as an assassin, but I’m always not good at it. Later, my teammates suggested that I should be bolder and more adventurous. Under the guidance and practice, I gradually became proficient (M12, 18-year-old).

I am an untalkative person in life, but through playing games, I try to actively communicate with my teammates and gradually become more cheerful and good at communication (F4, 19-year-old).

#### 3.1.3. Gain achievement and self-realization

Contrary to the harsh real society, the virtual game world demonstrates a new relatively equal social environment in its unique way and rich content. College students can explore multiple identities by experiencing multiple avatars or changing the appearance of avatars, thereby creating a virtual self-image that is sometimes compensatory and even restorative. Following are typical quotes from gamers who said that the game can bring them positive feelings.

I may be an ordinary person in life, but being a core character in games brings confidence (M1, 20-year-old).

In the virtual world of online games, college students can present themselves without the constraints of the single evaluation system of academic performance in real life. It provides the best arena for college students to gain achievement and fulfill their needs.

Sometimes I feel inferior because of my poor academic performance, and my classmates often treat me with colored glasses… Ranking as a king in the game, many players are willing to team up with me, which gives me a sense of accomplishment and brings back the confidence I lost in real life.

### 3.2. Satisfying the need for social life

#### 3.2.1. Broaden the social network

College students can make friends around the world with common interests in the virtual world. The game’s forum provides the means to initiate a chat or team invitations to skilled players, as well as the option to team up with players in the same city. Through this avenue, strangers can converse with each other across geographical distances and blur cultural boundaries. Following are typical quotes from gamers who widened the breadth of interpersonal communication.

I spend most of my time playing games with my real friends, sometimes accepting game invitations from strangers and meeting skilled friends (F4, 19-year-old).

Because of the game, I met people of different ages and professions who taught me about game operations (M6, 19-year-old).

#### 3.2.2. Increase social interaction

Online games offer everyone a convenient way to socialize and reduce social costs. The majority of participants reported that they would choose “Glory of Kings” as a common hobby to better integrate into the surrounding circle of friends. And some students will consciously take some compensatory actions against their friends who do not play games to maintain their friendship. Following are typical quotes from gamers who widened the breadth of interpersonal communication.

Playing games with unfamiliar individuals could quickly promote mutual understanding (F7, 19-year-old; M4, 21-year-old).

Playing games together can promote communication between roommates and increase common language (F2, 19-year-old).

I have a friend who does not play games and I tried to teach her to play but she refused. So I would study or eat with her after finishing the game time, and our relationship has always been tight (F5, 19-year-old).

#### 3.2.3. Value teamwork

In the game “Glory of Kings,” college students work as a team to promote friendship and learn from each other’s strengths. Cooperation is necessary for the progress of this game, and there is also a respondent who says he has transformed from a solitary player to a competent collaborator. Every interviewee agreed that they preferred to work with the team and were willing to do their best to cooperate with their teammates when playing the game and even sacrifice for teammates when necessary. Following are typical quotes from gamers who enjoyed teamwork.

Sometimes I could not figure it out on my own, but a team could succeed (M1, 20-year-old).

I used to fight alone when I think that just be happy with myself. But gradually I fell in love with playing in team battles and was happy to cooperate with the team. Meanwhile, if I do not focus on team cooperation, there will be no communication in the game, and the road to promotion will be bumpy (M3, 19-year-old).

### 3.3. Promoting academic performance

#### 3.3.1. Improve learning ability

The topic of academics was constantly mentioned and excavated in the interviews. The interviewees mentioned that online games helped them to master some transferable skills, such as problem-solving ability, quick thinking ability, etc. These abilities are usually trained unconsciously in games, and the player is able to experience the corresponding ability improvements in the real world when the match is over. Following are typical quotes from gamers who improved their ability to learn.

By studying the characters’ skill matching and line-ups in the game, my mind seemed to be more flexible and my problem-solving efficiency improved (M8, 18-year-old).

In order to improve my game skills, I watched the live video of the game host and learned their skills. As time passed, I found it easier to follow the teacher’s explanation in class while I could not keep up with the teacher’s quick thinking before (F2, 19-year-old).

#### 3.3.2. Increase interest in learning

The combination of abstract and complex knowledge with online games can change the current passive input education model and make students more interested and effective in learning. Following are typical quotes from gamers who said that they could accept the teaching content more effectively in the process of playing games.

The teacher introduced the historical relationships between different characters using the heroic characters from “Glory of Kings,” which suddenly made a sleepy history lesson come to life (F2, 19-year-old).

Some interviewees also proposed that combining the virtual currency obtained in the game with the correct rate of answering questions in the virtual classroom of college students can improve students’ classroom performance. Following are typical quotes from gamers who proposed that the mechanics of the game can be realized in the real world.

Using this way of learning can motivate me to arrange my online game time rationally, reduce my dependence on online games and enjoy studying more (F6, 20-year-old).

## 4. Discussion

This study examines whether “Glory of Kings” has a positive impact on college students. The qualitative nature of this study allows players to fully articulate specific aspects of their perspectives and allows researchers to analyze their views and attitudes in depth.

### 4.1. The impact on personal growth

Most participants in our study believe that the pressure in their university life can be relieved through online games which are consistent with the findings of Ari et al. (2020). The pressure from various aspects, such as academics, family, and social lives, as well as the confusion about the future, bring contemporary college students under more psychological pressure. Online games make it a way for college students to escape from reality and vent their suppressed emotions, and some students also accompany the relief method of verbal catharsis in the process. These findings are similar to those of Pine et al. (2020) that college students enjoy the happiness that accompanies the release of stress when gaming.

College students are in an important period of strengthening their self-consciousness system. As a medium, “Glory of Kings” provides players with a competitive platform whose ultimate goal is victory. In order to continue to win, players need to constantly reflect and summarize (Kow, 2017). Larsen (2020) proposed seven aspects of the skill theory framework to guide players to improve their skill level in the game. Therefore, players can correspond to the theoretical framework and reflect on their shortcomings and deficiencies. By having the opportunity to overcome shortcomings and reflect on themselves in the game world, ultimately a clearer self-awareness can be projected and benefit the player in the real world.

According to Maslow’s hierarchy of needs theory, the ultimate human need is self-actualization. Likewise, college students need to be respected and expect to gain achievement (Zhong and Yao, 2013). Liao et al. (2017) proposed that people will form virtual personalities on the Internet through self-remodeling, while virtual personalities are often different from the real world. In real life, the differences in economic conditions, living areas, material conditions, status, and the increasingly fierce competition environment often limit college students’ achievement experience. In online games, the boundary between physical reality and virtual reality will be blurred by more personalized and immersive environments (Young, 2009; Soutter and Hitchens, 2016; Kuo et al., 2017), resulting in a convenient way for college students to get achievements. Like the ordinary student M8, he was appreciated by others for his superb gaming skills. In conclusion, achieving self-worth and reaching potential are the goals to meet the need for self-actualization (Liao et al., 2017).

### 4.2. The impact on interpersonal communication

In the case of “Glory of Kings,” which is a confrontation that unfolds by grouping, the system offers both the opportunity to form teams on one’s own and the option of random matching. Online games try to expand player interactivity and social friendships in the setting of game rules to attract more players, which coincides with the needs of college students to interact with people (Ducheneaut and Moore, 2004). Linked to the trait of awakened autonomy among college students, they will establish emotional communication with like-minded friends they meet in “Glory of Kings,” and will also subjectively choose whom to team up with, which may be teammates they meet through the game or friends in the real world. The game provides a more comfortable communication platform for strangers in real life, thus widening the breadth of interpersonal communication.

Most of the participants in this research also tend to play “Glory of Kings” with their friends. According to the study by Croes and Antheunis (2021), people’s intimacy is directly proportional to the frequency of interaction. Consistent with the conclusion of Lai and Fung (2019), college students will not alienate their friends and feel lonely because of excessive investment in playing games. On the contrary, the emotions and experiences shared in the virtual game world can strengthen the bond between them (Granic et al., 2014). College students can fight side by side with their friends in the gaming world, and discuss gaming skills together offline. The game does not destroy or isolate their relationships. On the contrary, the tacit cooperation and communication between players in the game make the relationship closer.

Most of our participants recognize that they liked the teamwork model of “Glory of Kings.” Consist to the study by Chen and Chang (2020), a MOBA mobile game, it simulates a real-world situation in which temporary teams of strangers complete complex tasks in a short period. It can provide players with a unique platform for teamwork in such scenarios where social relationships need to be established quickly. It is further suggested in the study of Ewoldsen et al. (2012) that this can increase pro-social behaviors outside of the game environment, such as social and civic activities. It follows that teams derive satisfaction from existence, which is part of the meaning of collective effort. Choosing the group and cooperating to reach the same goals is precisely what is essential in real life as well.

### 4.3. The impact on academic performance

Contrary to conventional beliefs that playing games is intellectually lazy and sedating, playing games is shown to promote a wide range of cognitive skills (Granic et al., 2014). Compared with non-gamers, gamers show faster and more accurate attention allocation, and higher spatial resolution enhanced mental rotation in visual processing, these skills are transferred to other spatial tasks outside the game context (Green and Bavelier, 2012). In addition, scholars speculate that problem-solving skills can also be developed. On the whole, there are many good principles of learning built into good online games (Adachi and Willoughby, 2013), which could be applied to school learning tomorrow.

For this generation that grows up with the Internet, online games are an integral medium of communication and learning and have great potential for schools and workplaces to increase engagement, creativity and lifelong learning skills (Gee, 2003; Turkay and Adinolf, 2012; Granic et al., 2014). From the existing overall analysis results, the condition of the online games has more positive learning effect than traditional teaching condition. In addition to improving learning and memory (Sitzmann, 2011; Wouters et al., 2013), online gameplay has the potential to motivate individuals to participate in educational settings to improve students’ interest in learning (Clark et al., 2016).

### 4.4. Suggestions for parents and college management

By considering both the negative effects and potential benefits of the existence of games, many scholars proposed some balanced perspectives on the use of games for real-world personal growth with the intervention and supervision of a third party. The influence of the family is a pivotal factor, as it contributes significantly to the socialization of adolescents (Liu et al., 2015). Parental regulation through restrictive mediation or conversational mediation in adolescents’ gaming is one important factor that may limit adolescents’ gaming behavior (Colasante et al., 2022).

On the other hand, it is unscientific for parents to blindly prohibit their children from online games (Chen et al., 2020). More and more parents accept the Internet and games as valuable learning tools (Willoughby, 2008). Those parents help children become consumers to judge the advantages of games, plan a variety of leisure activities, mediate violent temptation games, and help children find the meaning of online games through positive communication (Chiu et al., 2004). Indeed, co-playing games with parents are associated with heightened prosocial behavior for girls (Coyne et al., 2011). In addition, researchers suggest that adolescents who receive the correct family education for the online game may learn ways to meet basic needs and self-control (Griffiths and Meredith, 2009; Liu et al., 2015; Chen et al., 2020). Parents should create a harmonious family atmosphere by continuously improving their parenting skills and building close relationships with their children, which is in line with the suggestions of Chiu et al. (2004). Meanwhile, parents should keep in touch with teachers to understand their children’s confusion as well as their use of the Internet at school to actively cooperate with the school’s policies.

Schools are digital education providers and prevention centers. Our results suggested that educators can take advantage of game-based education to facilitate problem-solving ability and to increase the study interests of college students (Whitton and MacLure, 2017). Moreover, universities or colleges can use online games as a potentially useful and beneficial educational tool to promote students’ positive emotions. For example, a school in Seoul, South Korea, set up an online game course that covers the humanities of games, game terminology, game manipulation, Q&A with professional players, and game science to positively impact students at different grades and schools in public education sites (Choi and Bang, 2021).

Many colleges and universities in China nowadays create electronic competitive social organizations and used network games in their daily teaching, not only to enrich students’ extracurricular life, but also to provide a platform for college students to find like-minded friends to play online games reasonably. Furthermore, some universities or colleges combine health education of online games with ideological and political work and try to establish college students’ mental profiles to understand their overall psychological conditions when they are playing online games. More strategies are needed to maximize the positive impact of online games on college students and help them grow healthily.

## 5. Implication

Despite the negative perception of online games in the Chinese cultural context, our study re-examines the impact of online games from the gamer’s perspective. To a certain extent, online games meet the personalized requirements of college students’ personal growth in Chinese collectivist culture, realize the need for the social interaction satisfaction, and enhance creativity in learning (Chen and Chang, 2020). The higher education nowadays should more scientifically guide teachers and parents to change their attitudes toward online games and recognize the benefits of online games (Whitton and MacLure, 2017). Meanwhile, the advantages of online games can be exploited to benefit more students by promoting their problem-solving ability through game-based education, contributing to more productive physical and mental health and learning (Granic et al., 2014).

## 6. Limitation

Although this study shows that “Glory of Kings” has a positive impact on contemporary college students, it should be noted that the data are cross-sectional and longitudinal studies are still needed to confirm the long-term impact of “Glory of Kings” on college students. Secondly, the data is only derived from the feedback that respondents actively self-reported, which means that there may be some hidden part of the self-reflection content of respondents. Thirdly, relevant quantitative studies can be carried out to further verify and analyze the results of this study.

## 7. Conclusion

In this study, it is found that meeting the need for personal growth, satisfying the need for social life and promoting academic performance are the main positive effects of playing online games. Some suggestions that enhance the supportive role of online games are structured for family and college education.

## Data availability statement

The original contributions presented in this study are included in the article/supplementary material, further inquiries can be directed to the corresponding authors.

## Ethics statement

The studies involving human participants were reviewed and approved by the Ethics Committee of Wenzhou Medical University (2022-028). Written informed consent was obtained from the individual for the publication of any potentially identifiable images or data included in this article.

## Author contributions

FL and DZ contributed to the conceptualization and writing—original draft. SW was in charge of the data collection and analysis. RZ assisted in data collation and analysis as well as literature search. CD and JZ contributed to the writing, reviewing, and editing. All authors contributed to manuscript revision, read, and approved the submitted version.
